# Florida-California Cancer Health Equity Center (CaRE^2^) Community Scientist Research Advocacy Program

**DOI:** 10.1007/s13187-023-02351-3

**Published:** 2023-08-29

**Authors:** B. Hensel, N. Askins, E. Ibarra, C. Aristizabal, I. Guzman, R. Barahona, B. Hazelton-Glenn, J. Lee, Z. Zhang, F. Odedina, D. J. Wilkie, M. C. Stern, L. Baezconde-Garbanati, S. Suther, F. Webb

**Affiliations:** 1 Department of Biobehavioral Nursing Science, University of Florida, Orlando, FL USA; 2 Department of Research and Graduate Programs, Florida State University, Orlando, FL USA; 3 Department of Population and Public Health Sciences, Keck School of Medicine of USC, University of Southern California, Los Angeles, CA USA; 4 USC Norris Comprehensive Cancer Center, Los Angeles, CA USA; 5 Institute of Public Health, Florida Agricultural and Mechanical University, Tallahassee, FL USA; 6 Department of Biostatistics, University of Florida, Gainesville, FL USA; 7 Mayo Clinic Comprehensive Cancer Center, Jacksonville, FL USA; 8 Department of Biobehavioral Nursing Science, University of Florida, Gainesville, FL USA; 9 Department of Surgery, University of Florida, Jacksonville, FL USA

**Keywords:** Community engagement, Research advocacy, Community scientist, Community Scientist Research Advocacy Program (CSRA), Health disparities

## Abstract

The Community Scientist Program (CSP), a model connecting researchers with community members, is effective to inform and involve the general population in health-related clinical research. Given the existing cancer disparities among Black/African American and Hispanic/Latino/a populations, more models describing how *cancer-related* CSPs are designed, implemented, and evaluated are needed. The Florida-California Cancer Research, Education and Engagement (CaRE^2^) Health Equity Center is a tri-institutional, bicoastal center created to eliminate cancer health disparities among Black/African American and Hispanic/Latino/a populations living in California and in Florida. The CaRE^2^ Center created a Community Scientist Research Advocacy (CSRA) training program for community members to become cancer research advocates. The CSRA program is currently a 13-week program conducted 100% virtually with all materials provided in English and Spanish for participants to learn more about prostate, lung, and pancreas cancers, ongoing research at CaRE^2^, and ways to share cancer research throughout their communities. Participants attend didactic lectures on cancer research during weeks 1–5. In week 4, participants join CSRA self-selected groups based on cancer-related topics of interest. Each group presents their cancer-related advocacy project developed during weeks 5–12 at the final session. In this paper, we describe the CaRE^2^ Health Equity Center’s CSRA program, share results, and discuss opportunities for improvement in future program evaluation as well as replication of this model in other communities.

**Supplementary Information:**

The online version contains supplementary material available at 10.1007/s13187-023-02351-3.

## Introduction

Community Scientist Programs (CSPs) have been valuable in increasing community engagement, knowledge, and awareness of research, trust in scientific research, and the quality of research conducted. CSPs help train community advocates in cancer research, providing them with necessary tools to help bridge the gap between scientists and the community at large. Trained community scientists can contribute to cancer research by serving on advisory boards, disseminating information, helping recruit participants, and serving as a bi-directional voice of the community in research. The application of a CSP model within diverse communities is relatively recent with a small body of peer-reviewed literature describing programs that share evidenced-based information about cancer prevention, screening, and treatment, tailored to populations at-risk for cancer [1, 2]. The existing evidence shows that outreach and education programs on cancer prevention, screening, and treatment are helpful in educating community members about the risks and approaches involved with various cancers [3]. Sharing information with the broader population of individuals who are at-risk but not yet diagnosed with cancer, in and throughout our communities, can be challenging, due to negative attitudes about screening modalities and fear of cancer diagnoses. In addition, it can be challenging to engage the community at large in cancer research, due to negative attitudes about research, and ineffective communication about how and why it is beneficial for them to participate in research as healthy volunteers [4]. Therefore, developing and implementing CSPs to bridge the gap between researchers and diverse populations at increased risk of developing and dying from cancer related diseases is a public health priority. CSP graduates will facilitate dissemination of cancer research information and participation of diverse communities in cancer research so that they can be represented in novel discoveries. Implementing cancer-related CSPs in California (CA) and in Florida (FL) is of high importance given existing cancer-related health disparities coupled with the large population of Black/African American (B/AA) and Hispanic/Latino/a (H/L) persons living in these states. In 2022, 7.1% (2.8 million) and 39% (15.6 million) of the CA population identified as (B/AA) or H/L, respectively; whereas in FL, 18.2% (3.8 million) and 25.6% (5.66 million) of the population identified as either B/AA or H/L. Altogether, these two states have greater representation of H/L compared to the entire US population (18.2%), and FL has greater proportion of B/AA compared to the entire USA (14.1%) [5–7]. Importantly, CA and FL ranked first and second in the USA respectively, for both annual number of new cancer cases and cancer deaths [8]. Moreover, cancer is the leading cause of death among the H/L population, accounting for 20% of deaths [9], whereas the B/AA population have the highest death rate and shortest survival of any racial/ethnic group in the USA for most cancers [9].

Dissemination of cancer research and participation in cancer research among B/AA and H/L populations present several challenges, including structural barriers to participation (e.g., cost of time involved in participation; culturally inefficacious communication strategies; insufficient access to medical insurance/facilities; transportation barriers; and issues related to child care, culture, and fear), fatalistic attitudes about cancer prognosis, and mistrust of clinical trial and healthcare systems [10, 11]. Therefore, CSP uniquely tailored to these two communities offer great promise for impact.

There is an increase in community-level engagement models being used [12] to create social and behavioral change on a community level, for example, training models for physicians and community health workers (CHWs) [13–15]. Newer models seek to train other key stakeholders such as patient advocates [16], who can share their voices, or “advocate” for cancer research, prevention, and treatment [16] which has contributed to a more patient-centered, patient-valued approach to cancer treatment. And there are also research advocates who serve as (1) a link between patients and scientific research, (2) help patients understand scientific information and research findings that may help them, (3) help researchers understand patient perspectives on research activities as members of scientific advisory boards and committees, (4) teach patients and their families about clinical trials, and (5) recruit patients to clinical trials [17]. Research advocates with training in the complex field of clinical research make powerful agents against misunderstanding, misinformation, and mistrust. Therefore, research advocates can be a key connection between individuals within the community and scientists.

With these ideas and needs in mind, we developed the CaRE^2^ Health Equity Center Community Scientist Research Advocacy (CSRA) training program, with the goals of informing, educating, and empowering community members to become cancer research advocates in FL and CA. The CSRA program is modeled after a successful educational program from the American Association for Cancer Research (AACR), the Scientist→Survivor Program (SSP) [18], and on the *Diffusion of Innovation Theory* (DIT) [19], which postulates that the more information is shared/disseminated, the more likely it is believed and eventually enacted. Evidence shows that crucial facilitators to effective cancer education and cancer research recruitment require communication modalities that are culturally appropriate, validation by members of the community’s social network as credible and trustworthy, and strong motivational messaging (i.e., why participation is beneficial *to them*) [20–23]. Our CSRA program has the goal of training these facilitators to create and sustain avenues to dismantle barriers between researchers and research advocates within our communities. In this study, we describe the CaRE^2^ Health Equity Center’s CSRA program, share results from the 2022 CSRA cohort, and discuss CSRA’s utility as a model for cancer research advocacy. Implications and opportunities for improvement are discussed to assist with program evaluation for future cohorts and replicating this model in other communities with culturally similar demographics as CA and FL where the CSRA program was developed and implemented.

## Methods

### Program Goals

Funded by the National Cancer Institute (NCI), the Florida-California Cancer Research, Education and Engagement (CaRE^2^) Health Equity Center is a tri-institutional, bicoastal center focused on eliminating prostate, lung, and pancreatic cancer health disparities among B/AA and H/L populations living in FL and in CA through research, education, and community outreach [24]. The purpose of the CaRE^2^ CSRA program is to inform, educate, and empower community members to become cancer research advocates in FL and CA. The program mission is to develop the manpower for research advocates who can work closely with cancer scientists to address cancer health disparities. The overall objectives of the CSRA program are to increase manpower for cancer research advocacy; strengthen the network of cancer research advocates; and increase multidirectional communication between cancer advocates with cancer survivors, community members, academic scientists, and policymakers, based on a model for community member advocacy for cancer research [18]. We report data for the 2022 CSRA program training cohort (March 21, 2022, to June 13, 2022).

### Recruitment, Expectations, and Outcomes

#### Recruitment

Our CSRA team recruited participants through a promotion flyer stating, “what you will learn,” program start date, how to apply, program details, contact information, and payment for participants ($1,000). Applications were promoted and received through the CaRE^2^ Health Equity Center’s website (https://care2healthequitycenter.org/community-scientist-program/), the CaRE^2^ Health Equity Center’s Community Reports, the center’s tri-institutional 15-member community advisory board, and the broader community. Interested individuals could directly apply on the website or contact any listed team member for more information or assistance with applying.

#### Program Expectations and Outcomes

Participants were expected to (1) attend weekly 2-hour virtual sessions for 13 weeks, (2) complete a self-guided curriculum (assigned videos and reading materials), (3) attend didactic lectures, and (4) implement a cancer research advocacy project within their communities. Appropriate for today’s society, advocacy projects were expected to align with a specific cancer disparity B/AA or H/L communities, prioritizing our CaRE^2^ Health Equity Center’s research focus on prostate, pancreas, and lung cancer. Learning objectives were to (1) discuss three ways that research advocacy is important to improving cancer health equity; (2) illustrate two examples of ethical cancer research activities that were observed during the experiential training; (3) determine the relevance of experience sharing and communications between advocates, students, and scientists to the quality of cancer research; and (4) disseminate research advocacy experiences through presentations. Participants received the $1,000 payment upon program completion of 80% attendance, project presentation, and submission of program evaluation.

### Curriculum, Structure, and Materials

#### Curriculum and Structure

CSRA is a 13-week program meeting 2 hours weekly and simultaneously implemented in CA and FL using a virtual format. CSRA participants worked on projects in groups of 3–4 individuals with a CaRE^2^ Center mentor assigned to each project group. The CRSA program included a self-guided learning curriculum, where the first 1–5 weeks included assigned reading with didactic lectures (Table 1), which included faculty from our tri-institutional center to include center core leaders and investigators who are experts in the selected fields of study. Participants used the information from each session and the self-guided curriculum to develop and implement a cancer research advocacy project in their communities.

**Table 1:** List of program didactic lecture topics

Topic	High Level Summary
IRB, Ethics, and Clinical Trials	- Historical studies and current protections set by federal government - Definitions and examples for ethical conduct of research
Cancer epidemiology	- Cancer risk factors and epidemiological approaches - Incidence and mortality of most prevalent cancers - Cancer health outcomes in B/AA and H/L populations living in CA and in FL
Prostate Cancer	- Prevention, screening recommendations and treatment - Body awareness: Prostate Cancer, Prostate Specific Antigen (PSA) vs Prostate Cancer Antigen 3 (PCA3) - CaRE^2^ research investigations
Cervical Cancer	- Know your family health history - Everything you always wanted to know about Human papillomavirus infection (HPV), but were afraid to ask
Breast Cancer	- The Multi-Ethnic Breast Cancer Survivorship Program
Laryngeal Cancer	- Exploring the role of biology in racial disparate clinical outcomes
Social Determinants of Health (SDOH)	- Definitions and examples of SDOH - Factors associated with cancer screening, treatment, and recovery
Community Engagement	- Engagement and maximizing participation - Definitions and community engagement continuum - Model definitions: Community-based participatory research (CBPR) and Community Engagement Research (CEnR)

#### Program Materials

All participants received the following: a program directory with contact information for each participant to stimulate group project communication; a project PowerPoint template; cancer research materials on pancreas, prostate, lung, and breast cancer from various accredited cancer sites; project brainstorming guide; advocacy training guide; and a CaRE^2^ CSRA handbook (can be viewed in supplemental materials). All materials were provided both in English and Spanish, and the Zoom sessions had simultaneous translation by attending translators. Our CSRA team communicated weekly with participants via email to provide updates and access to materials for each session. A shared Google Drive was used for bidirectional sharing of all materials and session recordings, which allowed participants to have continued access and to collectively work as a team.

The remaining nine weeks were focused on helping participants identify an advocacy project; create their teams; research information; design, implement, and evaluate advocacy projects; and report findings, experiences, and lessons learned from conducting the advocacy project. Participants gave a 15-minute presentation on their advocacy project at the final session (week 13).

#### Advocacy Project

Participants were asked to learn about and promote an aspect of the CaRE^2^ Health Equity Center research and/or cores within the community through a research advocacy project. The requirements included the following: (1) feature work of the CaRE^2^ Center cores and/or research; (2) share information via mass and/or social media; and (3) use of reach and impact measures. The goals to be achieved through the advocate-mentor relationship included the following: (1) community scientist advocate understands the research project such as project aims, recruitment, data collection, data analysis, and dissemination; (2) mentor understands and contributes to all phases such as planning, implementation, and evaluation of the research advocacy project; and (3) community scientist presents results on a research advocacy project designed for community dissemination. Participants and mentors met weekly and participated in one training session while working in smaller groups to complete a cancer research advocacy project for presentation at the end of the program.

The advocacy projects were presented via a Zoom webinar to CaRE^2^ Health Equity Center stakeholders, which included the CaRE^2^ network of community members, scientists, and advocates interested in cancer research, cancer healthcare, and research disparities. Community Scientist Research Advocates were able to participate in a practice presentation session to receive feedback to strengthen their final presentations. A presentation template was provided to program participants to aid in presentation development.

### Program Evaluation

Participants were asked to complete three (3) surveys that were developed by our team to evaluate knowledge, program evaluation, and program feedback. All program evaluation surveys were offered in English and Spanish. Qualtrics was utilized to create and disseminate surveys.

#### Knowledge

Knowledge was assessed after presentations were completed throughout weeks 1–5 regarding the ethical conduct of research as well as for various types of cancers including, but not limited to, prostate, pancreas, breast, and cervical cancers. For each question, responses were grouped into “correct” or “other.” Responses of “unknown” and “incorrect” were classified as “other.” Knowledge questions were developed based on presenters’ lectures/webinars. Questions were provided by presenters and program planners and were created directly from presentations and lectures given during the first 4 weeks of the program. Participants’ knowledge and perceptions of the CSRA program were evaluated at the start (week 1; pre), at 6 weeks (mid), and at the end (week 13; post) of the program. Participants who did not complete the program were not asked to complete post-surveys assessing knowledge since they did not attend all lectures/webinars.

#### Expectations

Participants received pre- and post-surveys, which asked about CSRA program expectations and feedback on ways to improve our CSRA program (see supplemental materials for participant feedback). Question responses were grouped into “agree” and “other.” Responses of “agree” and “strongly agree” were classified as “agree,” and “disagree” and “strongly disagree” were classified as “other.”

### Statistical Analysis

All analyses were performed using R Statistical Software (v4.2.3; R Core Team 2023). The program evaluation surveys at pre-, post-, and mid-program employed a cross-sectional design, without matching participants. Participants pre- and-post responses to questions were analyzed to assess knowledge (correct vs. other) reported at the start of the program and at the mid-point of the program compared to the end of the program, using Fisher’s exact test for the correct answers. Similarly, program expectations (agree vs. other) were compared between unmatched pre-and-post participants, using Fisher’s exact test. Percentages of correct answers for aggregated questions (questions 1–5 and 8–9) for each participant were summarized with mean and standard deviation and compared between two-time point surveys, using Wilcoxon rank sum test.

## Results

All applicants (*N* = 20) who applied to the 2022 CaRE^2^ Health Equity Center CSRA program were accepted; their demographic characteristics are summarized in Table 2. Among them, 13 (68.4%) participants self-identified as B/AA and 7 (35.0%) participants identified as H/L. A total of 18 (90.0%) participants self-identified as female and 2 (10.0%) self-identified as male. The majority (*N* = 11; 55.0%) had college degrees with 8 (40.0%) reporting some college and 1 (5.0%) being a high school graduate. The majority (65.0%) of participants were from CA (Los Angeles County [*N* = 13]), with 30% being from FL (Duval County [*N* = 1], Leon County [*N* = 3], Orange County [*N* = 2]), and 5% from Georgia (Gwinnett County [*N* = 1]). Participants reported learning about our program by the following methods: Twitter, community organizations, or referrals from a CaRE^2^ Center member, family, and/or friend. The program launched on March 21, 2022, with 18/20 participants who met the requirement of attending at least 80% (10 weeks) of virtual training sessions. All of the CSRA participants identified areas for advocacy, resulting in at least 4 working groups that examined breast, lung, pancreas, or prostate cancer.

**Table 2:** Demographical characteristics of program participants

Characteristic	Pre, *N* = 20
Highest Education	
College degree Some college but no degree High school graduate	11 (55.0%) 8 (40.0%) 1 (5.0%)
Ethnicity (Hispanic/Latino/a)	
Yes No	7 (35.0%) 13 (65.0%)
Race	
Black/African American White Other Unknown	13 (68.4%) 5 (26.3%) 1 (5.3%) 1 (5.3%)
Gender	
Female Male	18 (90.0%) 2 (10.0%)
Residence	
California Florida Georgia	13 (65.0%) 6 (30.0%) 1 (5.0%)

### Program Evaluation: Knowledge

There were 20 responses at the start of the program, whereas 16 responses were received at mid- and post-program due to attrition of two participants and no response from two other participants. Overall, across all knowledge questions, participants had a mean (SD) of 67.9 (7.9) at pre-survey and a mean of 71.4 (9.0) at post-survey (*p* = 0.2). Participants had a mean (SD) score on knowledge questions of 60.0 (14.6) when assessed mid-way through the program and a mean score of 68.2 (11.0) when assessed at the end of the program (post-survey) (*p* = 0.13). Tables 3 and 4 includes responses and percentages to each question. We observed a non-significant increase in knowledge-based questions, as seen in the tables.

**Table 3:** Pre- and post-survey comparison: Knowledge

Characteristic: Ethical Research	Pre, *N* = 20	Post, *N* = 16	*p* Value
One of the three principles of ethics in research is “Respect – treating each person as a free individual with dignity.” An example of respect is:			> 0.9
Correct Other	19 (95.0%) 1 (5.0%)	15 (93.8%) 1 (6.2%)	
An example of the principle of “Justice” or the duty to be fair in research is:			> 0.9
Correct Other	19 (95.0%) 1 (5.0%)	16 (100.0%) 0 (0.0%)	
Both researchers and community scientist must follow research protocol.			1
Correct	20 (100.0%)	16 (100.0%)	
The principle of beneficence in research means that research should benefit the participants and society without any risks.			0.3
Correct Other	1 (5.0%) 19 (95.0%)	3 (18.8%) 13 (81.2%)	
Research informed consent forms should state that the participant has the right to withdraw at any time without penalty			> 0.9
Correct Other	19 (95.0%) 1 (5.0%)	16 (100.0%) 0 (0.0%)	
Which of these is a social determinant of health? Pick all that apply.			0.7
Correct Other	17 (85.0%) 3 (15.0%)	12 (75.0%) 4 (25.0%)	
What are two prevention approaches of sociodeterminants? Pick all that apply.			0.2
Correct Other	0 (0.0%) 20 (100.0%)	2 (13.3%) 14 (87.5%)	
*Past Engagement and Awareness*			
Have you attended presentations (i.e., lectures, webinars) on community engagement (CE)?			1
Correct	20 (100%)	16 (100%)	
Where should community engagement (CE) take place?			1
Correct	20 (100%)	16 (100%)	

Other=Combined incorrect/unknown answers. This analysis shows correct vs. other

**Table 4:** Mid- and post-survey comparison: Knowledge

Characteristic	Mid, *N* = 16	Post, *N* = 16	*p* Value
*Prostate Cancer*			
African American men are more likely to develop prostate cancer than Caucasian men			> 0.9
Correct Other	15 (93.8%) 1 (6.2%)	16 (100.0%) 0 (0.0%)	
What does the prostate do?			> 0.9
Correct Other	15 (93.8%) 1 (6.2%)	16 (100.0%) 0 (0.0%)	
What is prostate cancer?			> 0.9
Correct Other	14 (87.5%) 2 (12.5%)	15 (93.8%) 1 (6.2%)	
A high PSA number means you have prostate cancer			0.5
Correct Other	5 (31.2%) 11 (68.8%)	8 (50.0%) 8 (50.0%)	
At what age should a man ask his doctor about prostate cancer screening?			> 0.9
Correct Other	7 (43.8%) 9 (56.2%)	7 (43.8%) 9 (56.2%)	
*Cervical Cancer*			
What is human papillomavirus (HPV)?			0.7
Correct Other	6 (37.5%) 10 (62.5%)	8 (50.0%) 8 (50.0%)	
How many types of HPV are known?			0.7
Correct Other	11 (68.8%) 5 (31.2%)	9 (56.2%) 7 (43.8%)	
What percentage of people will get a genital HPV infection in their lifetime?			0.2
Correct Other	10 (62.5%) 6 (37.5%)	14 (87.5%) 2 (12.5%)	
What diseases are caused by HPV? Choose all that apply			0.7
Correct Other	11 (68.8%) 5 (31.2%)	9 (56.2%) 7 (43.8%)	
What is the HPV preventative vaccine name?			> 0.9
Correct Other	15 (93.8%) 1 (6.2%)	15 (93.8%) 1 (6.2%)	
*Breast Cancer*			
What percentage of deaths in women are due to metastatic breast cancer?			0.14
Correct Other	3 (18.8%) 13 (81.2%)	8 (50.0%) 8 (50.0%)	
What is circulating tumor DNA?			0.5
Correct Other	14 (87.5%) 2 (12.5%)	16 (100.0%) 0 (0.0%)	
What population of women are less likely to not be diagnosed with early-stage breast cancer?			0.4
Correct Other	10 (62.5%) 6 (37.5%)	13 (81.2%) 3 (18.8%)	
How many women with invasive breast cancer develop a metastatic recurrence?			0.5
Correct Other	8 (50.0%) 8 (50.0%)	11 (68.8%) 5 (31.2%)	
What does micrometastatic disease (MRD) stand for?			
Correct Other	8 (50.0%) 8 (50.0%)	8 (50.0%) 8 (50.0%)	> 0.9
*Laryngeal Cancer*			
Survival rate of laryngeal cancer is strongly associated with what? Choose all that apply			> 0.9
Correct Other	0 (0.0%) 16 (100.0%)	1 (6.2%) 15 (93.8%)	
What percentage of head and neck cancers are classified as squamous cell carcinomas?			0.5
Correct Other	6 (37.5%) 10 (62.5%)	9 (56.2%) 7 (43.8%)	
What is a biorepository?			0.5
Correct Incorrect	14 (87.5%) 2 (12.5%)	16 (100.0%) 0 (0.0%)	
What type of samples are collected and stored in a biorepository? Choose all that apply			0.002
Correct Other	0 (0.0%) 16 (100.0%)	8 (50.0%) 8 (50.0%)	
*Family History*			
Which of the following are associated with family health history? Choose all that apply			0.7
Correct Other	8 (50.0%) 8 (50.0%)	6 (37.5%) 10 (62.5%)	
Why is family health history important?			1
Correct	16 (100.0%)	16 (100.0%)	
What are the four (4) different environments?			> 0.9
Correct Other	3 (18.8%) 13 (81.2%)	2 (12.5%) 14 (87.5%)	
How can you collect your family health history?			> 0.9
Correct Other	15 (93.8%) 1 (6.2%)	15 (93.8%) 1 (6.2%)	
What are red flags for hereditary cancers?			> 0.9
Correct Other	12 (75.0%) 4 (25.0%)	11 (68.8%) 5 (31.2%)	
Three or more family members are affected by cancer. What risk category is this?			0.5
Correct Other	14 (87.5%) 2 (12.5%)	16 (100.0%) 0 (0.0%)	

Other=Combined incorrect/unknown answers. This analysis shows correct vs. other

Participants’ knowledge regarding aspects of prostate cancer such as whether African American men are more likely to develop prostate cancer, what does the prostate do, what is prostate cancer, as well as the meaning of a high prostate-specific antigen (PSA) test was higher at the end of the program (i.e., post responses) compared with responses midway through the program (Table 4). Whereas participants’ responses at the end of the program were also higher for human papillomavirus (HPV) knowledge, regarding knowing what HPV is, and the percentage of people who will get genital herpes, participants’ knowledge was lower at program end compared to midway regarding how many types of HPV are known, and the causes of HPV. Participants’ knowledge also increased at the end of the program regarding breast cancer. For example, the percentage of participants knowing the correct percentage of deaths in women due to metastatic breast cancer, what is circulating tumor Deoxyribonucleic Acid (DNA), and how many women with invasive breast cancer develop metastatic disease was higher at program end compared with midway (Table 4).

The percentages of participants answering correctly about laryngeal cancer, squamous cell carcinomas, as well as the purposes and types of biorepositories used to collect samples increased by program end with significant increases observed for knowing what types of samples are collected and stored in biorepositories. On the other hand, the percentages of participants answering correctly on questions related to Social Determinants of Health (SDOH) were lower at program end compared with the percentages of participants answering correctly on SDOH questions midway through the program (Table 4).

### Program Evaluation: Expectations

We used a Likert scale to evaluate program expectations, which resulted in a mean (SD) of 94.0 (8.8) of participants who “agreed” that they were satisfied with the program at the start compared to a mean (SD) of 91.0 (12.9) of participants who “agreed” that they were satisfied with the program at the end (overall *p* = 0.5). Overall, the percentages at the end of the program were not statistically significant compared to at the start of the program. Shown in Table 5, the following showed increases in strongly agree and/or no change comparing end versus start of the program: “believing that advocates play an important role” (100% vs. 95%); “community between advocates and academic cancer researchers is underappreciated” (70% vs.65%); “I believe that patient advocacy is essential to improving cancer health equity” (100% vs. 100%); and “as a result of my participation, I believe I will be a better advocate” (100% vs. 100%). On the other hand, the percentages of participants who strongly agreed to the following decreased and/or had no change by program end compared with program start: “benefited from participating” (95% vs.100%); “gained new knowledge about cancer” (95% vs. 100%); “academic researchers will benefit from sharing my experience” (95% vs. 100%); “I have benefited from learning more about research activities” (90% vs. 100%); and “I have improved my ability to be an advocate” (100% vs. 100%). All percentages are rounded to the nearest tenth.

**Table 5:** Pre- and post-survey comparison: Program Expectations

Characteristic	Pre, *N* = 20	Post, *N* = 20	*p* Value
Benefited from participating in 2022 Community Scientist Research Advocacy Program			> 0.9
Agree Other	20 (100.0%) 0 (0.0%)	19 (95.0%) 1 (5.0%)	
I gained new knowledge about cancer research			> 0.9
Agree Other	20 (100.0%) 0 (0.0%)	19 (95.0%) 1 (5.0%)	
I know that academic researchers will benefit from sharing my cancer experiences			> 0.9
Agree Other	20 (100.0%) 0 (0.0%)	19 (95.0%) 1 (5.0%)	
I believe that cancer advocates play an important role in the quality of cancer research			> 0.9
Agree Other	20 (100.0%) 0 (0.0%)	19 (95.0%) 1 (5.0%)	
As a cancer advocate, I have benefited from learning more about research activities			0.5
Agree Other	20 (100.0%) 0 (0.0%)	18 (90.0%) 2 (10.0%)	
Communication between cancer advocates and students is underappreciated			> 0.9
Agree Other	15 (75.0%) 5 (25.0%)	14 (70.0%) 6 (30.0%)	
Communication between cancer advocates and academic cancer researchers is underappreciated			> 0.9
Agree Other	13 (65.0%) 7 (35.0%)	14 (70.0%) 6 (30.0%)	
I believe that patient advocacy is essential to improving cancer health equity			1
Agree	20 (100.0%)	20 (100.0%)	
As a result of participating in the 2022 Community Scientist Research Advocacy Program, I have improved my ability to be an advocate			1
Agree	20 (100.0%)	20 (100.0%)	
As a result of participating in the 2022 Community Scientist Research Advocacy Program, I will be able to play a greater role in helping my community understand the importance of participation in cancer research/clinical trials			1
Agree	20 (100.0%)	20 (100.0%)	

Agree=Combined agree/strongly agree answers. Other=Combined disagree/strongly disagree. This analysis shows agree vs. other

## Discussion

We present implementation, evaluation results, and lessons learned for our first bicoastal, bilingual CSRA training program, conducted virtually in CA and FL. Whereas the CRSA program was initially designed as a hybrid virtual and in-person program, with experiential training and poster presentations, though, due to the COVID-19 pandemic, we were forced to implement it fully virtual, which enabled the simultaneous training of advocates in CA and FL. The CaRE^2^ Health Equity Center CSRA program was successful in increasing the number of trained community members to become advocates for cancer research. Altogether, to date, a total of 26 community members completed the CSRA program sessions in 2019 and 2022; and we will be graduating another cohort of 20 participants in August 2023.

For 2022, as a group, participants’ overall knowledge about cancer and cancer research changed positively, although changes were not statistically significant. We also observed a smaller proportion of the group with knowledge accuracy at the end of program compared to start, which was unexpected but may have related to loss of participants completing the unmatched post-survey. Similarly, the overall level of expectations at the start of the program changed, but not in a statistically significant manner, with some questions showing decreases at the end of program. One explanation for knowledge decreases or change in expectations by program end compared to program start and midway is that participants failed to recall what was learned several weeks prior. It also suggests that the questions were not optimally matched to the program content, which focused on ongoing research rather than cancer incidence, prevalence and mortality, screening, diagnosis, and treatment practices. Overall, these results suggest that we need to re-think the questions used to assess knowledge to better capture the unique content they are gaining in the program, as well as program expectations. Amy Leader [17] points out that research programs have few measures for evaluating basic science/laboratory collaborations and more are focused on population science/public health interventions. Among those that do include metrics on research advocacy impact, most measure long-term outcomes and scientific impact, not the process of research advocacy or evaluation on community member impact. For these reasons, the CaRE^2^ Center creates a research culture where the inclusion, training, and support of cancer research advocates are conducted and evaluated with the same priority as any other center activity, in the way of a true partnership.

Our CSRA model has several innovative aspects compared to other cancer community scientist programs [15–17]. Having participants complete an “advocacy project” was purposefully designed to (a) increase participants’ self-efficacy to be an advocate with “hands-on”/active planning and (b) leverage the CaRE^2^ Health Equity Center’s reach, impact, and sustainability where advocates design and implement a project on cancer research being conducted within the center. Another innovation was the program delivery, with simultaneous, synchronous, and bilingual implementation in CA and in FL, focusing on two racial and ethnic populations. To meet the needs of these two populations, all content (e.g., didactic lectures and program materials) was in English and Spanish via translated materials or using synchronous translators, thus allowing monolingual Spanish-speaking participants (*N* = 2) to fully participate. Also innovative was the fact that the program was implemented in a 100% virtual environment, which allowed for each participant to interact and subsequently present their cancer research advocacy projects bicoastally in FL and CA. Offering the CSRA program through a virtual environment allowed for increased participation and program completion across the three institutions’ bicoastal locations and allowed for interactions between cancer advocates across the two coasts. Finally, we highlight the innovation that the CSRA program faculty and staff provided mentorship and guidance to teams as they worked to complete their advocacy projects, such as inviting experts, promoting their advocacy projects, and identifying required resources from evidence-based resources (i.e., NCI, American Cancer Society).

Key strengths of the program include recruitment of participants that exceeded expectations, participation of guest speakers who presented on cancer research  related topics, repeated evaluations focused on knowledge, and program expectations throughout the program. Importantly, all participants agreed that they would make a greater impact in their communities given their participation in the CSRA program. Moreover, the CSRA program advocates successfully implemented their advocacy projects. For example, two webinars with both CA and FL participant collaboration included *Understanding Pancreatic Cancer* (June 2022) and *Breast Cancer Research* (July 2022)*.* Since our 2022 program, a program alumnus was selected to be a Breast Cancer Research Advocate for Susan G. Komen in Washington, DC, in June 2023 at the Advocacy Summit on Capitol Hill.

We recognize the opportunity to reassess our curriculum and evaluation processes to see a clearer trend within our program results about the overall program training approach. We recognize that a cohort of 20 participants is a limited number of respondents to detect significant differences in knowledge or program effectiveness and that our knowledge questions need to be more rigorous to avoid a ceiling effect at the beginning of the program, which reduces the potential to show a significant increase. Unfortunately, the cohort size is limited by grant funds and available team members. To achieve greater survey rigor, we will ask our didactic lecture speakers to provide learning objectives/goals and questions for their presentations, add questions related to our self-guided learning materials, and provide each participant with a code number to identify responses over time. Finally, we will assess each participant’s application, such as education level and primary language, to assure we are tailoring the content to the correct audience and using better targeted survey questions.

Implementation of the CSRA program was met with several challenges. For example, the COVID-19 pandemic introduced setbacks given that we were not able to offer the planned in-person experience in CaRE^2^ Center labs at respective institutions. Switching to a virtual platform required a learning curve among participants on how to use Zoom, including use by our Spanish translators. In addition, internet or Wi-Fi sometimes failed for CSRA program participants and faculty. Other challenges included time management across three bicoastal institutions in different time zones. Despite these challenges, the CaRE^2^ Health Equity Center CSRA Program is a unique and effective health promotion model where participants learn about cancer research. They also gain skills in planning, developing, and implementing an advocacy project, as well as presenting projects to their community members and cancer scientists. Another setback of our program results is attrition and/or not completing the surveys, which affected the analysis of the pre-knowledge survey (*N* = 20) and post-knowledge survey (*N* = 16). Moving forward, we plan to measure longitudinal reach and impact of CSRA alumni through biannual contact that assesses advocates’ ongoing cancer-related activities and involvement resulting from CSRA program completion.

We have taken our lessons learned to restructure our program for the new 2023 cohort, in hopes for a more accurate measurement of increase in knowledge and program expectations. We have additionally taken into consideration participant feedback from our program evaluation survey. As we evaluate our program further, we continue to include our Community Advisory Board in participant selection, as well as biostatistics experts in our ongoing program planning. To strengthen our program, we look to add the involvement of program alumni to help with feedback and evaluation for our program. These planned changes are consistent with the increased attention to patient public involvement (PPI) in research design and recruitment (i.e., gatekeepers) in cancer programs, which we may find useful as our program continues to develop and is evaluated each year [25, 26].

A key recommendation for replication of our program is emphasizing the need to tailor the program to the population in the corresponding catchment area. This includes the use of simultaneous session translators, material translations, and materials that accompany the training, as well as consideration of literacy level of participants. Other recommendations include, effective evaluation methods, advance program planning, time management, and a well-versed implementation team. Our program was bicoastally implemented, which takes successful coordination as well as considering the program teams’ different time zones. We recommend obtaining materials from an accredited institution(s) or organization(s) via online, in-person, or mail, such as but not limited to the NCI or American Cancer Society, that best serves your program structure and participants.

In summary, we present an innovative community scientist research advocacy training program, uniquely developed for B/AA and H/L communities with a focus on disseminating information about cancer health disparities in these communities. We achieved our goal to train a workforce of trained advocates that can serve as a bidirectional bridge between cancer scientists and the community at large. Our program can be adapted to serve the needs of different communities.

### Supplementary Information

Below is the link to the electronic supplementary material.

Supplementary file1 (PDF 101 KB)

Supplementary file2 (PDF 264 KB)

Supplementary file3 (PDF 319 KB)
