# Supplementary material for: Florida-California Cancer Health Equity Center (CaRE2) Community Scientist Research Advocacy Program
Source: J Cancer Educ. 2023 Aug 29;38(5):1429–39. doi: 10.1007/s13187-023-02351-3 (PMC10509126; doi:10.1007/s13187-023-02351-3)
Supplement: Supplementary file 1 — Supplementary file1 (PDF 101 KB) [file 13187_2023_2351_MOESM1_ESM.pdf]

Florida-California Cancer Health Equity Center (CaRE^2) Community Scientist Research Advocacy Program

Jornal of Cancer Education

Hensel, B.[1]; Askins, N.[2]; Ibarra, E.[3,4]; Aristizabal, C.[3,4]; Guzman, I.[1]; Barahona, R.[3,4]; Hazelton-Glenn, B.[5]; Lee, J.[6], Zhang, Z.[6]; Odedina, F.[7]; Wilkie, D.J.[1]; Stern, M. C.[3,4]; Baezconde-Garbanati, L.[3,4]; Suther, S.[5], Webb, F.[8].

Affiliations

University of Florida, Department of Biobehavioral Nursing Science, Gainesville, FL, USA[1]; Florida State University, Department of Research and Graduate Programs, Orlando, FL, USA[2]; University of Southern California, Department of Population and Public Health Sciences, Keck School of Medicine of USC, Los Angeles, CA, USA[3]; USC Norris Comprehensive Cancer Center, Los Angeles, CA, USA[4]; Florida Agricultural and Mechanical University, Institute of Public Health, Tallahassee, FL, USA[5]; University of Florida, Department of Biostatistics, Gainesville, FL, USA[6]; Mayo Clinic Comprehensive Cancer Center, Jacksonville, FL, USA[7] University of Florida, Department of Surgery, Jacksonville, FL, USA[8];

Corresponding Author

Brooke Hensel, MS, CHES: bhensel@ufl.edu, 407-313-7112, 6550 Sanger Road, Orlando, FL, 32827

Program completers provided several responses with suggestions for program improvement:

- “You do great work. Maybe make future opportunities for the advocates to engage in research through sending them open opportunities for such via email. Create a database of investigators looking for advocates/community partners/stakeholder for projects or community engagement events (speaking engagements).”
- “I think the program is great and afforded me the opportunity to expand”
- “Really great program and helpful to be an advocate for our community”
- “Great program, I loved the diversity of backgrounds and education levels throughout the groups.”
- “The Citizen Scientist Advocacy Program was a good investment of time and as a seasoned Advocate I was able to glean a few nuggets along the way. Having to put together the PowerPoint presentation was a practical assignment. However, I was looking to learn how research is done from a scientist perspective.”
- “Loved it. Information and presenters were great. Good information. Looking forward to next year.”
- “Suggest teams share their contact information at the beginning, Suggest the requirements are clear on the booklet vs what is shared at the meetings because it can be confusing at times. Suggest to check the survey questions for some had errors and were a little confusing. However, overall I think it is a good program with good opportunities.”
- “When I signed up for CaRE2 I wasn't quite sure what to expect, or if I would even get selected to participate, but now I am so grateful for the opportunity it presented my fellow group members, and myself to learn about how Community Advocacy works. All of the guest speakers had so much knowledge and information to share about a wide variety of topics…Having everything available in Google Drive to watch at another time is very helpful, because I felt like during the week I was so consumed with work I did not have time until the weekend to review things, and for some of my group who were still going through active treatment, it was hard for them to find the energy to engage…It was a privilege to continually have feedback on how our presentation was coming along towards the last 3 weeks from the instructors, and it would be good to hear what our fellow classmates thought of each group and their topics...”
- “1. Make sure that everyone has a working web camera and working microphone throughout the program. So, they can participate in a equal amount time in the presentation. We’re going to get a $1,000 each. Effort, time and contribution should be included to determine if everyone gets the stipend. 2. A group evaluation for everyone. 3. An outline of PowerPoint presentation set up. Dr. Webb and Rosa suggested to add animation and color. Greatly

appreciated. Yes, did add it. But maybe tell others too. 4. The communication from the staff should be the same. Is was a little confusing with different answers.”
