# Supplementary material for: Florida-California Cancer Health Equity Center (CaRE2) Community Scientist Research Advocacy Program
Source: J Cancer Educ. 2023 Aug 29;38(5):1429–39. doi: 10.1007/s13187-023-02351-3 (PMC10509126; doi:10.1007/s13187-023-02351-3)
Supplement: Supplementary file 2 — Supplementary file2 (PDF 264 KB) [file 13187_2023_2351_MOESM2_ESM.pdf]

Florida-California Cancer Health Equity Center (CaRE[2]) Community Scientist Research Advocacy Program

Journal of Cancer Education

Hensel, B.[1]; Askins, N.[2]; Ibarra, E.[3,4]; Aristizabal, C.[3,4]; Guzman, I.[1]; Barahona, R.[3,4]; Hazelton-Glenn, B.[5]; Lee, J.[6], Zhang, Z.[6]; Odedina, F.[7]; Wilkie, D.J.[1]; Stern, M. C.[3,4]; Baezconde-Garbanati, L.[3,4]; Suther, S.[5], Webb, F.[8].

Affiliations

University of Florida, Department of Biobehavioral Nursing Science, Gainesville, FL, USA[1]; Florida State University, Department of Research and Graduate Programs, Orlando, FL, USA[2]; University of Southern California, Department of Population and Public Health Sciences, Keck School of Medicine of USC, Los Angeles, CA, USA[3]; USC Norris Comprehensive Cancer Center, Los Angeles, CA, USA[4]; Florida Agricultural and Mechanical University, Institute of Public Health, Tallahassee, FL, USA[5]; University of Florida, Department of Biostatistics, Gainesville, FL, USA[6]; Mayo Clinic Comprehensive Cancer Center, Jacksonville, FL, USA[7] University of Florida, Department of Surgery, Jacksonville, FL, USA[8];

Corresponding Author

Brooke Hensel, MS, CHES: bhensel@ufl.edu, 407-313-7112, 6550 Sanger Road, Orlando, FL, 32827

Florida-California Cancer Research Education and Engagement (CaRE²) Health Equity Center

Community Scientist Research Advocacy Program

Program Handbook

March 2022

Welcome to CaRE^2!

We are excited that you have accepted our invitation to complete CaRE^2 Health Equity Center's Community Scientist Research Advocacy Program. As Community Scientist research advocates, together we will make a positive impact to improve the relevance, conduct and impact of cancer research in our communities.

Program Overview

The purpose of the CaRE^2 Community Scientist Research Advocacy Program is to inform, educate and empower community members, like you, to become cancer advocates in Florida and California. Primary objectives are to increase manpower for cancer research advocacy; strengthen the network of cancer research advocates; and increase multi-directional communication between cancer advocates with cancer survivors, community members, academic scientists and policy makers.

We have designed an interactive program to include self-guided reading, experiential learning, and mentoring opportunities that finish with each team presenting their own advocacy project. Given the COVID-19 pandemic, we have modified this program for 100% delivery in a virtual environment. Information for all program materials are in this Program Handbook, and will be reviewed at orientation/during the first week of the program.

Participant Expectations

Participants are expected to attend weekly sessions, complete self-guided curriculum and implement an advocacy project. Each participant will have help to develop a team that is best suited to design/plan and implement an advocacy project appropriate for today's society (i.e., target audience, social distancing).

Upon completion, Community Scientist Research Advocates (CSRAs) will be able to:

1. Discuss three ways that cancer research and community involvement can improve cancer health equity
2. Develop an advocacy project on CaRE^2 cancer research
3. Present the advocacy project to key stakeholder groups to include community members, scientists and policy makers

Participants will also receive a $1,000 honorarium upon program completion and submission of the program evaluation.

Curriculum

Funded by the National Institutes of Health (NIH) National Cancer Institute (NCI), the CaRE^2 Health Equity Center is an integrative center with six cores and three active research studies. We will share more information about CaRE^2 cores, projects and opportunities for you to conduct a cancer advocacy project. We also provide webinars to increase understanding of factors at play in the world of cancer health disparities research.

Cancer Research Advocacy Project

Each participant will be asked to develop an advocacy project focused on cancer research. The purpose of the advocacy project is to promote some aspect of the CaRE^2 research center and or projects with our community. Requirements of advocacy projects: 1) feature work of the CaRE^2 center cores and/or research 2) share information via mass and/or social media, to 3) include reach and impact measures.

We have designed weekly sessions to promote organic development of advocacy projects and match you to mentors who can guide/contribute to your project development.
We hope the following is achieved through the advocate-mentor relationship:

- Community Scientist Research Advocate understands the research project such as project aims, recruitment, data collection, data analysis, and dissemination.
- Mentor understands and contributes to all phases (planning, implementation, evaluation) of the advocacy project.
- Community Scientist presents results on an advocacy project designed for community dissemination.

Community Scientist Virtual Symposium

The program will culminate in a virtual symposium where each participant presents his/her advocacy project. This presentation will be held as a webinar over Zoom that will be aimed at CaRE^2 Health Equity Center stakeholders to include the CaRE^2 network of community members, scientists and advocates interested in cancer research and eliminating cancer health care and research disparities. Community Scientists are encouraged to participate in a practice presentation session scheduled June 6 to give and receive feedback to strengthen their final presentation.

A presentation template will be provided to trainees/Community Scientists research advocates to aid in presentation development. Information to include:

- Background on Community Scientist’s advocacy work
- Project’s purpose/goal
- Description of CaRE^2 area or component featured in project
- Community of Focus: What population is the project designed to reach?
- Project Implementation: What was done? How was it done? Where was it done?
- Project Results: How many people were reached? Who was impacted? How many people were impacted
- Lessons Learned & Next Steps: What did we learn? What are the next steps?
- Specific examples of how Community Scientist’s advocacy experiences can be leveraged to strengthen the CaRE^2 advocacy project’s service of the community.

Program at a Glance

The program is from March 21, 2022 through June 13, 2022. The first four weeks will include self-guided reading along with nightly webinars to learn more about $CaRE^2$. We have structured the curriculum this way so that you receive as much information prior to week 5 when you start to develop your project. Planning for the advocacy project will begin in week 4 where we meet at times that are most convenient for everyone in the group. Projects are presented in the Community Scientist Virtual Symposium scheduled for the program day in June 2022.

After the first two weeks, weekly webinars might be changed to another time most convenient for the group. We have also included a list of optional webinars that are offered as part of the $CaRE^2$ programs. The log-in information for whenever we meet is provided below and will also be distributed via an emailed calendar invite.

Join Zoom meeting	https://ufl.zoom.us/j/93732603195?pwd=a21sa21BcG5na1VzMEhONWlyMVNhUT09
Join by phone	US: +1 301 715 8592 or +1 312 626 6799 or +1 646 558 8656 or +1 253 215 8782 or +1 346 248 7799 or +1 669 900 6833
Meeting ID	937 3260 3195
Password	831072
Meeting Time:	Mondays from 3:00 - 5:00 PM PST/ 6:00 - 8:00 PM EST

Attendance: In order to successfully complete this program participants are expected to:

- Attend at least 10 (≥ 80%) weekly sessions of the 13-week program
- Actively communicate with identified mentor(s) to seek their input/guidance
- Develop and implement an enrichment advocacy project
- Summarize and report findings from advocacy project
- Present findings and lessons learned in the Community Scientist Virtual Symposium
- Participate in continuous quality improvement evaluations

Honorarium

The $CaRE^2$ Health Equity Center will provide a $1,000 honorarium to participants who successfully complete this program. The Community Scientist honorarium of $1,000 will be disbursed to the account you provide to each university's fiscal department. Participants will follow the disbursement guidelines from each university. The disbursement will be upon successful completion of the program on June 13, 2022. In order to ensure timely disbursement of your honorarium, we will help you complete all of the required forms to be approved by our universities by March 30, 2022.

The following team program team members are available to assist you with completing and submitting the required forms to be approved in the university's system:

- Name and email address of FAMU contact is: TBD
- Name and email address of USC contact is: Eduardo Ibarra, ibarrae@usc.edu
- Name and email address of UF contact is: Brooke Hensel, bhensel@ufl.edu

Community Scientist Advocacy Program

Week #	Session Dates	Topics	Weekly Activities/Assignments
1	March 21, 2022	Program Orientation & CaRE^2 Overview (Together)<br>Confirm registration with university's fiscal office<br>Self-Learning Curriculum CaRE^2 Center/ Project Description<br>Advocacy Project Template<br>Center Announcements/Calendar of Events<br>Self-Evaluation Advocacy Checklist	Start self-learning curriculum
2	March 28, 2022	IRB, Ethics & Clinical Trials: *Dr. Sandra Suther*<br>Cancer Epidemiology: *Dr. Mariana Stern*	Continue self-learning curriculum<br>Attend webinars
3	April 4, 2022	Center research: Prostate Cancer PSA vs PCA3: *Dr. Renee Reams*<br><br>Ice Breaker/Project Discussion	Continue self-learning curriculum<br>Attend webinars
4	April 11, 2022	Cancer Research - pilot projects*: Dr. Luisel Ricks-Santi*<br>Cancer research project: *Dr. Bodour Sahlia, Dr. Martin Kast*<br>Advocacy Project Opening Discussion	Attend webinar
5	April 18, 2022	Social Determinants of Health (COC): *Dr. Lourdes Baezconde-Garbanati*<br>Engagement and Maximizing Participation (COC): *Dr. Fern Webb*<br>Cancer research project: *Dr. Kristianna Fredenburg*<br><br>Discuss Program Assessments and select topics<br>Discuss advocacy project: progress, challenges & next steps	Create presentation of proposed project
6	April 25, 2022	Drafting and discussing project details	Present Proposed Projects<br>Work on advocacy project<br>Create advocacy team/ mentors
7	May 2, 2022	Status- update on project proposal/implement	Continue working on advocacy project<br>Identify & contact potential mentors
8	May 9, 2022	Status- update on project implementation	Continue working on advocacy project<br>Share project progress
9	May 16, 2022	Status- update on project implementation<br>Discuss report/presentation	Present progress on advocacy project<br>Incorporate feedback
10	May 23, 2022	Status- update on project implementation and report<br><br>Practice final report/presentation	Continue working on advocacy project<br>Share project progress
11	May 30, 2022	**Memorial Day - No Session** - Can meet with group to work on project if desired	Finish advocacy project<br>Determine steps for completion
12	June 6, 2022	Present final report/presentation<br>Provide evaluation/discuss program impact and opportunities for improvement	Finalize/complete project<br>Draft/prepare final presentation
13	June 13, 2022		Present enrichment advocacy project

CaRE^2 Mentoring Network includes individuals who lead or conduct cancer research as part of CaRE^2**.** The following table includes a brief description of mentors' research interest along with their email address, which is the best way to reach them.

**Name**	**Site**	**Research Interests**	**Email**
Carolina Aristizabal, MD, MPH, CHES	USC	Community Based Participatory Research, Cancer Health Disparities and Minority Health	caristiz@usc.edu
Brooke Hensel, MS, CHES	UF	Research Coordinator, Community Outreach Team	bhensel@cop.ufl.edu
Lourdes Baezconde-Garbanati, PhD, MPH	USC	Community Based Participatory Research, Cancer Health Disparities and Minority Health	baezcond@usc.edu
Kristianna Fredenburg, PhD	UF	Assistant Professor	kfredenburg@ufl.edu
Eduardo Ibarra	USC	Project Specialist	ibarrae@usc.edu
Fayetta Justin	FAMU	Administrative Coordinator	Fayetta.Justin@hcahealthcare.com
Luisel Ricks-Santi, PhD	UF	Associate Professor	lrickssanti@cop.ufl.edu
Sandra Suther, PhD	FAMU	Public Health Genomics, Medical Anthropology, Qualitative Research Methods, Cultural Competency, Planning and Evaluation of Health Programs, Maternal and Infant Health, Socio-behavioral and Health Communication Theory	sandra.suther@famu.edu
Mariana Stern, PhD	USC	Molecular Epidemiology	marianas@usc.edu
Fern J. Webb, PhD	UF	Community-based health interventions, community engagement research	fern.webb@jax.ufl.edu
Renee Reams, PhD	FAMU	Prostate Cancer	renee.reams@famu.edu
Bodour Salhia, PhD	USC	Breast Cancer	salhia@usc.edu
W. Martin Kast, PhD	USC	Microbiology and Immunology	Martin.Kast@med.usc.edu

Community Scientist Advocacy Program
Self-Guided Reading Material

**CaRE² Center**	CaRE² Health Equity Center:<br>Care2 Center – Florida-California Health Equity Center (care2healthequitycenter.org)
**Engagement and Maximizing Participation**	Community Engagement: An Introduction: https://www.youtube.com/watch?v=AAU-vK8cBtg<br><br>Lesson 2: Inspiring Community Participation to Solve Local Problems:<br>https://www.youtube.com/watch?v=OSqDOi82sdQ<br><br>The Secret to Community Engagement:<br>https://www.youtube.com/watch?v=i-fbl2C-dKc<br><br>Moving from Services to Advocacy: Maximizing the Role of Community Health Workers in Policy, System, and Environmental Changes:<br>https://chwcentral.org/blog/moving-services-advocacy-maximizing-role-community-health-workers-policy-systems-and
**Cancer Epidemiology**	What is Epidemiology?: https://www.youtube.com/watch?v=r9poHB-ldqk<br><br>Introduction to Cancer Epidemiology: https://www.youtube.com/watch?v=aBFADKBkKCw
**Social Determinants of Health**	Social Determinants of Health:<br>https://www.healthypeople.gov/2020/topics-objectives/topic/social-determinants-of-health<br><br>Addressing Social Determinants of Health:<br>https://www.youtube.com/channel/UCCsieriDUNU-2-nNmXngYAw/search?query=social+determinant
**IRB, Research Ethics, and Clinical Trials**	Henrietta Lacks, the Tuskegee Experiment, and Ethical Data Collection: Course Statistics # 12:<br>https://www.youtube.com/watch?v=CzNANZnoiRs<br><br>About Research Participation:<br>https://www.hhs.gov/ohrp/education-and-outreach/about-research-participation/index.html<br><br>How We Go From Animal Model to Clinical Trial:<br>https://www.youtube.com/watch?v=FXKGjqKFohw<br><br>The Clinical Trial Journey: https://www.youtube.com/watch?v=iWqQiJeP5ac
**Biobanking**	CTSI Biorepository:<br>https://www.ctsi.ufl.edu/research/laboratory-services/ctsi-biorepository-2/<br><br>USC Biorepository:<br>Virtual Repository – Care2 Center (care2healthequitycenter.org)<br><br>Biobanking of patient-derived cancer models for applied research and clinical application:<br>https://www.youtube.com/watch?v=QgiH_mvL2fM<br><br>Mayo Clinic Biobank: https://www.youtube.com/watch?v=a6qLBidtW-Y
**Omics**	Exploring Space Through You Series:<br>https://www.youtube.com/user/NASAgovVideo/search?query=omics
