# Supplementary material for: Florida-California Cancer Health Equity Center (CaRE2) Community Scientist Research Advocacy Program
Source: J Cancer Educ. 2023 Aug 29;38(5):1429–39. doi: 10.1007/s13187-023-02351-3 (PMC10509126; doi:10.1007/s13187-023-02351-3)
Supplement: Supplementary file 3 — Supplementary file3 (PDF 319 KB) [file 13187_2023_2351_MOESM3_ESM.pdf]

Florida-California Cancer Health Equity Center (CaRE^2) Community Scientist Research Advocacy Program

Journal of Cancer Education

Hensel, B.[1]; Askins, N.[2]; Ibarra, E.[3,4]; Aristizabal, C.[3,4]; Guzman, I.[1]; Barahona, R.[3,4]; Hazelton-Glenn, B.[5]; Lee, J.[6], Zhang, Z.[6]; Odedina, F.[7]; Wilkie, D.J.[1]; Stern, M. C.[3,4]; Baezconde-Garbanati, L.[3,4]; Suther, S.[5], Webb, F.[8].

Affiliations

University of Florida, Department of Biobehavioral Nursing Science, Gainesville, FL, USA[1]; Florida State University, Department of Research and Graduate Programs, Orlando, FL, USA[2]; University of Southern California, Department of Population and Public Health Sciences, Keck School of Medicine of USC, Los Angeles, CA, USA[3]; USC Norris Comprehensive Cancer Center, Los Angeles, CA, USA[4]; Florida Agricultural and Mechanical University, Institute of Public Health, Tallahassee, FL, USA[5]; University of Florida, Department of Biostatistics, Gainesville, FL, USA[6]; Mayo Clinic Comprehensive Cancer Center, Jacksonville, FL, USA[7] University of Florida, Department of Surgery, Jacksonville, FL, USA[8];

Corresponding Author

Brooke Hensel, MS, CHES: bhensel@ufl.edu, 407-313-7112, 6550 Sanger Road, Orlando, FL, 32827

Florida-California Educación y Participación en la Investigación del Cáncer (CaRE^2) Centro de Equidad en Salud

Programa de capacitación en defensa de los científicos comunitarios

Manual del Programa

Marzo 2022

¡Bienvenido a CaRE²!

Estamos entusiasmados de que haya aceptado nuestra invitación para completar el programa Capacitación en Defensa de Científicos Comunitarios de CaRE² Centro de Equidad en Salud. Como defensores de Científico Comunitariosco, juntos tendremos un impacto positivo para mejorar la relevancia, la conducta y el impacto de la investigación del cáncer en nuestras comunidades.

Descripción general del programa
El propósito del programa CaRE² Capacitación en Defensa de Científicos Comunitarios es informar, educar y empoderar a los miembros de la comunidad, como usted, para que se conviertan en defensores del cáncer en Florida y California. Los objetivos principales son aumentar la mano de obra para la defensa de la investigación del cáncer; fortalecer la red de defensores de la investigación del cáncer; y aumentar la comunicación multidireccional entre los defensores del cáncer con los sobrevivientes del cáncer, los miembros de la comunidad, los científicos académicos y los responsables políticos.

Hemos diseñado un programa interactivo para incluir la lectura autoguiada, el aprendizaje experiencial y las oportunidades de tutoría, que culminan con un proyecto de promoción. Dada la pandemia de COVID-19, hemos modificado este programa para una entrega de 90% en un entorno virtual y 1 dia en persona.

La información para todos los materiales del programa se encuentra en este manual del programa y se revisará en la orientación / durante la primera semana del programa.

Expectativas de los participantes
Se espera que los participantes asistan a sesiones semanales, completen un plan de estudios autoguiado e implementen un proyecto de promoción. Cada participante tendrá ayuda para desarrollar un equipo que se adapte mejor para diseñar / planificar e implementar un proyecto de defensa apropiado para la sociedad actual (es decir, público objetivo, distanciamiento social).

Al finalizar, Defensores de los Científicos Comunitarios (CSA) podrá:
1. Discutir las formas en que la investigación del cáncer y la participación de la comunidad pueden mejorar la equidad en la salud del cáncer
2. Desarrollar un proyecto de promoción de la investigación del cáncer CaRE²
3. Presentar el proyecto de promoción a los grupos de partes interesadas clave para incluir a miembros de la comunidad, científicos y responsables políticos

Los participantes también recibirán un honorario de $1,000 al finalizar el programa y enviar la evaluación del programa.

Programa de capacitación en defensa de los científicos comunitarios

Currículo

Financiado por el Instituto Nacional del Cáncer de los Institutos Nacionales de Salud (NIH / NCI), el Centro de Equidad en Salud $CaRE^2$ es un centro integrador con seis núcleos y tres estudios de investigación activos. Compartiremos más información sobre los núcleos, proyectos y oportunidades de $CaRE^2$ para que usted lleve a cabo un proyecto de defensa del cáncer. También ofrecemos seminarios web para aumentar la comprensión de los factores en juego en el mundo de la investigación de las disparidades de salud del cáncer.

Proyecto de Defensa de la Investigación del Cáncer

Se le pedirá a cada participante de que desarrolle un proyecto de defensa centrado en la investigación del cáncer. El propósito del proyecto de promoción es promover algún aspecto del centro de investigación $CaRE^2$ y/o proyectos con nuestra comunidad. Requisitos de los proyectos de promoción: 1) presentar el trabajo de los núcleos del centro $CaRE^2$ y / o la investigación 2) compartir información a través de las redes masivas y / o sociales, para 3) incluir medidas de alcance e impacto.

Hemos diseñado sesiones semanales para promover el desarrollo orgánico de proyectos de promoción y emparejarlo con mentores que pueden guiar / contribuir al desarrollo de su proyecto.
Esperamos que se logre lo siguiente a través de la relación defensor-mentor:

- Defensor del Científico Comunitario entiende el proyecto de investigación, como los objetivos del proyecto, el reclutamiento, la recopilación de datos, el análisis de datos y la difusión.
- Mentor entiende y contribuye a todas las fases (planificación, implementación, evaluación) del proyecto de promoción.
- Community Scientist presenta los resultados de un proyecto de promoción diseñado para la difusión comunitaria.

Simposio Virtual de Científicos Comunitarios

El programa culminará en un simposio virtual donde cada participante presentará su proyecto de incidencia. Esta presentación se llevará a cabo como un seminario web a través de Zoom que estará dirigido a los participantes de $CaRE^2$ Centro de Equidad en Salud para incluir la red $CaRE^2$ de miembros de la comunidad, científicos y defensores interesados en la investigación del cáncer y la eliminación de la atención médica del cáncer y las disparidades de investigación. Se alienta a los Científicos Comunitarios a participar en una sesión de presentación de práctica programada para el 6 de junio para dar y recibir comentarios para fortalecer su presentación final.

Se proporcionará una plantilla de presentación a los aprendices / defensores de Científicos Comunitarios para ayudar en el desarrollo de la presentación. Información por incluir:

- Antecedentes sobre el trabajo de defensa de Científicos Comunitarios
- Propósito/objetivo del proyecto
- Descripción del área o componente $CaRE^2$ presentado en el proyecto
- Comunidad de enfoque: ¿A qué población está diseñado llegar el proyecto?
- Implementación del proyecto: ¿Qué se hizo? ¿Cómo se hizo? ¿Dónde se hizo?
- Resultados del proyecto: ¿A cuántas personas se llegó? ¿Quién se vio afectado? Cuántas personas se vieron afectadas
- Lecciones aprendidas y próximos pasos: ¿Qué aprendimos? ¿Cuáles son los próximos pasos?
- Ejemplos específicos de cómo las experiencias de incidencia de Científico Comunitarios pueden aprovecharse para fortalecer el servicio de la comunidad del proyecto de defensa CaR

Programa de capacitación en defensa de los científicos comunitarios

Program de un vistazo

El programa es del 21 de Marzo de 2022 al 13 de Junio de 2022. Las primeras cuatro semanas incluirán lectura autoguiada junto con seminarios web nocturnos para aprender más sobre CaRE^2. Hemos estructurado el plan de estudios de esta manera para que reciba la mayor cantidad de información antes de la semana 5 cuando comience a desarrollar su proyecto. La planificación del proyecto de promoción comenzará en la semana 4, donde nos reuniremos en los momentos más convenientes para todos en el grupo. Los proyectos se presentan en el Simposio Virtual de Científicos Comunitarios del Cáncer programado para el día del programa en Junio de 2022.

Después de las primeras dos semanas, los seminarios web semanales pueden cambiarse a otro horario más conveniente para el grupo. También hemos incluido una lista de seminarios web opcionales que se ofrecen como parte de los programas CaRE^2. La información de inicio de sesión para cada vez que nos reunimos se proporciona a continuación y también se distribuirá a través de una invitación de calendario enviada por correo electrónico.

Únase a la reunión de Zoom	https://ufl.zoom.us/j/93732603195?pwd=a21sa21BcG5na1VzMEhONWIyMVNhUT09
Únete por teléfono	US: +1 301 715 8592 or +1 312 626 6799 or +1 646 558 8656 or +1 253 215 8782 or +1 346 248 7799 or +1 669 900 6833
ID de reunión	937 3260 3195
Contraseña	831072
Tiempo de reunión	Lunes de 3:00 - 5:00 PM PST/ 6:00 - 8:00 PM EST

Atención: Para completar con éxito este programa, se espera que los participantes:

- Asistir al menos 10 (≥ 80%) sesiones semanales del programa de 13 semanas
- Comunicarse activamente con los mentores identificados para buscar su opinión / orientación
- Desarrollar e implementar un proyecto de promoción del enriquecimiento
- Resumir e informar los hallazgos del proyecto de promoción
- Presentar los hallazgos y lecciones aprendidas en el Simposio Virtual de Científicos Comunitarios
- Participar en evaluaciones de mejora continua de la calidad

Honorarios

El Centro de Equidad en Salud CaRE^2 proporcionará honorarios de $1,000 a los participantes que completen con éxito este programa. Los honorarios de $1,000 de Cientificos Comunitarios se desembolsará en la cuenta que proporcione al departamento fiscal de cada universidad. Los participantes seguirán las pautas de desembolso de cada universidad. El desembolso se realizará al completar con éxito el programa el 13 de junio de 2022. Para garantizar el desembolso oportuno de sus honorarios, lo ayudaremos a completar todos los formularios requeridos para que sean aprobados por nuestras universidades antes del 30 de marzo de 2022.

Los siguientes miembros del equipo del programa están disponibles para ayudarlo a completar y enviar los formularios requeridos para ser aprobados en el sistema de la universidad:

- El nombre y la dirección de correo electrónico del contacto de FAMU son: TBD
- El nombre y la dirección de correo electrónico del contacto de USC son: Eduardo Ibarra, ibarrae@usc.edu
- El nombre y la dirección de correo electrónico del contacto de UF son: Brooke Hensel, bhensel@ufl.edu

Programa de capacitación en defensa de los científicos comunitarios

**Week #**	**Fechas** de las sesiones	**Temas**	**Actividades/Tareas Semanales**
1	21 de Marzo de 2022	Orientación del programa y descripción general de $CaRE^2$ (juntos)<br>Confirmar registro con la oficina fiscal de la universidad<br>Currículo de autoaprendizaje Centro $CaRE^2$/ Descripción del proyecto<br>Plantilla de proyecto de promoción<br>Anuncios del Centro/Calendario de Eventos<br>Lista de verificación de defensa de la autoevaluación	Comience el currículo de autoaprendizaje
2	28 de Marzo de 2022	IRB, Ética y Ensayos Clínicos<br>*Dra. Sandra Suther*<br>Epidemiología del cáncer: *Dra. Mariana Stern*	Continuar con el currículo de autoaprendizaje<br>Asistir a seminarios web
3	4 de Abril de 2022	Investigación del centro: cáncer de próstata PSA vs PCA3: *Dra. Renee Reams*<br><br>Discusión sobre el proyecto/rompehielos	Continuar con el currículo de autoaprendizaje<br>Asistir a seminarios web
4	11 de Abril de 2022	Investigación del Cáncer - proyectos piloto: *Dr. Luisel Ricks-Santi*<br>Proyecto de investigación del cáncer: *Dr. Bodour Sahlia, Dr. Martin Kast*<br>Discusión de apertura del proyecto de defensa	Asista a seminarios web
5	18 de Abril de 2022	Determinantes Sociales de la Salud (COC): *Dra. Lourdes Baezconde-Garbanati*<br>Compromiso y Maximización de la Participación (COC): *Dra. Fern Webb*<br>Proyecto de investigación del cáncer: *Dr. Kristianna Fredenburg*<br><br>Discutir las evaluaciones del programa y seleccionar temas<br>Discutir el proyecto de cabildeo: progreso, desafíos y próximos pasos	Crear la presentación del proyecto propuesto
6	25 de Abril de 2022	Redacción y discusión los detalles del proyecto	Presentar Proyectos Propuestos<br>Trabaja en proyecto de incidencia<br>Crear un equipo de defensa/mentores
7	2 de Mayo de 2022	Estado: actualización de la propuesta/implementación del proyecto	Continuar trabajando en proyecto de incidencia<br>Identifique y contacte a mentores potenciales
8	9 de Mayo de 2022	Estado: actualización de la implementación del proyecto	Continuar trabajando en proyecto de incidencia<br>Compartir el progreso del proyecto
9	16 de Mayo de 2022	Estado: actualización de la implementación del proyecto<br>Discutir informe/presentación	Presentar el progreso del proyecto de incidencia

Programa de capacitación en defensa de los científicos comunitarios

			Incorporar comentarios
10	23 de Mayo de 2022	Estado: actualización de la implementación del proyecto e informe<br><br>Práctica informe final/presentación	Continuar trabajando en proyecto de incidencia<br>Compartir el progreso del proyecto
11	30 de Mayo de 2022	Día de los Caídos - Sin sesión - Puede reunirse con el grupo para trabajar en un proyecto si lo desea	Terminar proyecto de incidencia<br>Determinar los pasos para completar
12	6 de Junio de 2022	Presentar informe/presentación final<br>Proporcionar evaluación/discutir el impacto del programa y las oportunidades de mejora	Finalizar/completar proyecto<br>Redactar/preparar la presentación final
13	June 13, 13 de Junio de 2022		Presentar proyecto sobre enriquecimiento de abogacía

CaRE² Mentoring Network incluye a personas que lideran o realizan investigaciones sobre el cáncer como parte de CaRE². La siguiente tabla incluye una breve descripción del interés de investigación de los mentores junto con su dirección de correo electrónico, que es la mejor manera de comunicarse con ellos.

Name	Site	Research Interests	Email
Carolina Aristizabal, MD, MPH, CHES	USC	Community Based Participatory Research, Cancer Health Disparities and Minority Health	caristiz@usc.edu
Brooke Hensel, MS, CHES	UF	Research Coordinator, Community Outreach Team	bhensel@cop.ufl.edu
Lourdes Baezconde-Garbanati, PhD, MPH	USC	Community Based Participatory Research, Cancer Health Disparities and Minority Health	baezcond@usc.edu
Kristianna Fredenburg, PhD	UF	Assistant Professor	kfredenburg@ufl.edu
Eduardo Ibarra	USC	Project Specialist	ibarrae@usc.edu
Fayetta Justin	FAMU	Administrative Coordinator	Fayetta.Justin@hcahealthcare.com
Luisel Ricks-Santi, PhD	UF	Associate Professor	lrickssanti@cop.ufl.edu
Sandra Suther, PhD	FAMU	Public Health Genomics, Medical Anthropology, Qualitative Research Methods, Cultural Competency, Planning and Evaluation of Health Programs, Maternal and Infant Health, Socio-behavioral and Health Communication Theory	sandra.suther@famu.edu
Mariana Stern, PhD	USC	Molecular Epidemiology	marianas@usc.edu
Fern J. Webb, PhD	UF	Community-based health interventions, community engagement research	fern.webb@jax.ufl.edu
Renee Reams, PhD	FAMU	Prostate Cancer	renee.reams@famu.edu
Bodour Salhia, PhD	USC	Breast Cancer	salhia@usc.edu
W. Martin Kast, PhD	USC	Microbiology and Immunology	Martin.Kast@med.usc.edu

Material de lectura autoguiado

**Centro CaRE^2**	CaRE^2 Health Equity Center:<br>Care2 Center – Florida-California Health Equity Center (care2healthequitycenter.org)
**Compromiso y maximización de la participación**	Participación de la comunidad: una introducción:<br>https://www.youtube.com/watch?v=AAU-vK8cBtg<br><br>Lección 2: Inspirar la participación de la comunidad para resolver problemas locales:<br>https://www.youtube.com/watch?v=OSqDOi82sdQ<br><br>El secreto de la participación comunitaria:<br>https://www.youtube.com/watch?v=i-fbl2C-dKc<br><br>Pasar de los servicios a la defensa: maximizar el papel de los trabajadores de salud comunitarios en los cambios de políticas, sistemas y ambientales:<br>https://chwcentral.org/blog/moving-services-advocacy-maximizing-role-community-health-workers-policy-systems-and
**Epidemiología del cáncer**	¿Qué es la epidemiología?: https://www.youtube.com/watch?v=r9poHB-ldqk<br><br>Introducción a la Epidemiología del Cáncer: https://www.youtube.com/watch?v=aBFADKBkKCw
**Determinantes sociales de la salud**	Determinantes sociales de la salud:<br>https://www.healthypeople.gov/2020/topics-objectives/topic/social-determinants-of-health<br><br>Abordar los determinantes sociales de la salud:<br>https://www.youtube.com/channel/UCCsieriDUNU-2-nNmXngYAw/search?query=social+determinant
**IRB, Ética de la Investigación y Ensayos Clínicos**	Henrietta Lacks, el experimento tuskegee y la recopilación de datos éticos: Estadísticas del curso # 12: https://www.youtube.com/watch?v=CzNANZnoiRs<br><br>Acerca de la participación en la investigación:<br>https://www.hhs.gov/ohrp/education-and-outreach/about-research-participation/index.html<br><br>Cómo pasamos del modelo animal al ensayo clínico:<br>https://www.youtube.com/watch?v=FXKGjqKFohw<br><br>El viaje de los ensayos clínicos: https://www.youtube.com/watch?v=iWqQiJeP5ac
**Biobancos**	Biorepositorio CTSI: https://www.ctsi.ufl.edu/research/laboratory-services/ctsi-biorepository-2/<br><br>Biobanco de modelos de cáncer derivados de pacientes para investigación aplicada y aplicación clínica: https://www.youtube.com/watch?v=QgiH_mvL2fM<br><br>Biobanco de Mayo Clinic: https://www.youtube.com/watch?v=a6qLBidtW-Y
**Ómica**	Explorando el espacio a través de ti Serie:<br>https://www.youtube.com/user/NASAgovVideo/search?query=omics
